# Physical Agent Modalities in Early Osteoarthritis: A Scoping Review

**DOI:** 10.3390/medicina57111165

**Published:** 2021-10-26

**Authors:** Giulia Letizia Mauro, Dalila Scaturro, Francesca Gimigliano, Marco Paoletta, Sara Liguori, Giuseppe Toro, Giovanni Iolascon, Antimo Moretti

**Affiliations:** 1 Department of Surgery, Oncology, and Stomatology, University of Palermo, 90133 Palermo, Italy; giulia.letiziamauro@unipa.it (G.L.M.); dalila.scaturro@unipa.it (D.S.); 2 Department of Physical and Mental Health and Preventive Medicine, University of Campania “Luigi Vanvitelli”, 80100 Naples, Italy; francesca.gimigliano@unicampania.it; 3 Department of Medical and Surgical Specialties and Dentistry, University of Campania “Luigi Vanvitelli”, 80100 Naples, Italy; sara.liguori@unicampania.it (S.L.); giuseppe.toro@unicampania.it (G.T.); giovanni.iolascon@unicampania.it (G.I.); antimo.moretti@unicampania.it (A.M.)

**Keywords:** osteoarthritis, early osteoarthritis, rehabilitation, physical therapy modalities, physical agents, electric stimulation therapy, pulsed electromagnetic field, extracorporeal shockwave therapy, vibration therapy

## Abstract

Early osteoarthritis (EOA) still represents a challenge for clinicians. Although there is no consensus on its definition and diagnosis, a prompt therapeutic intervention in the early stages can have a significant impact on function and quality of life. Exercise remains a core treatment for EOA; however, several physical modalities are commonly used in this population. The purpose of this paper is to investigate the role of physical agents in the treatment of EOA. A technical expert panel (TEP) of 8 medical specialists with expertise in physical agent modalities and musculoskeletal conditions performed the review following the PRISMA-ScR (Preferred Reporting Items for Systematic Reviews and Meta-Analyses Extension for Scoping Reviews) model. The TEP searched for evidence of the following physical modalities in the management of EOA: “Electric Stimulation Therapy”, “Pulsed Electromagnetic field”, “Low-Level Light Therapy”, “Laser Therapy”, “Magnetic Field Therapy”, “Extracorporeal Shockwave Therapy”, “Hyperthermia, Induced”, “Cryotherapy”, “Vibration therapy”, “Whole Body Vibration”, “Physical Therapy Modalities”. We found preclinical and clinical data on transcutaneous electrical nerve stimulation (TENS), extracorporeal shockwave therapy (ESWT), low-intensity pulsed ultrasound (LIPUS), pulsed electromagnetic fields stimulation (PEMF), and whole-body vibration (WBV) for the treatment of knee EOA. We found two clinical studies about TENS and PEMF and six preclinical studies—three about ESWT, one about WBV, one about PEMF, and one about LIPUS. The preclinical studies demonstrated several biological effects on EOA of physical modalities, suggesting potential disease-modifying effects. However, this role should be better investigated in further clinical studies, considering the limited data on the use of these interventions for EOA patients.

## 1. Introduction

Osteoarthritis (OA) is the most common degenerative and progressive joint disease, characterized by localized pain and impaired mobility, with relevant implications on both the quality of life of affected patients and socio-economic burden [1]. This condition is very challenging to manage, considering the extreme variability of clinical and instrumental findings of OA patients. Moreover, no available intervention can effectively counteract the structural changes of different tissues involved in the pathogenesis of OA, such as bone, synovium, and cartilage [2].

Much effort has been made to identify OA in the early stages to avoid the occurrence of major joint structural alterations. However, early OA (EOA) is a controversial condition due to the lack of an agreement on a unanimous definition, diagnosis, and, above all, of therapeutic intervention [3].

Several diagnostic criteria have been proposed for EOA. The first definition of EOA was provided by Luyten et al., stating that early knee OA (EKOA) is defined if three of the following criteria are met: two or more episodes of knee pain lasting more than 10 days, Kellgren and Lawrence (KL) grades 0–2, cartilage lesions in arthroscopy, magnetic resonance imaging (MRI) evidence of cartilage or meniscus damage, and/or bone marrow lesions (BMLs) of the subchondral bone [4]. More recently, Migliore et al. proposed diagnostic criteria of EKOA in patients over 40 years based on symptoms lasting for less than 6 months (knee pain without any recent trauma associated with joint stiffness), the presence of clinical risk factors (e.g., family history of OA, metabolic syndrome, malalignment and/or leg length discrepancy), and no radiological findings of OA (KL grade 0) [5]. Moreover, Luyten et al. proposed new EKOA classification criteria based on patient-reported outcomes (i.e., Knee Injury and Osteoarthritis Outcome score (KOOS) for defining pain and functional limitation), clinical examination (joint line tenderness and/or crepitus), and KL grade 0–1 [6].

Considering these uncertainties about a clear definition of EOA, various therapeutic approaches, both pharmacological and/or non-pharmacological, have been proposed in clinical practice, although no agreement has been reached on recommended interventions.

Pharmacological therapy (i.e., NSAIDs) is commonly prescribed to reduce pain and inflammation and might affect disease progression through immune-mediated mechanisms. Symptomatic slow-acting drugs, such as glucosamine, chondroitin sulfate, and diacerein, have also been suggested as early interventions for knee OA [7]. Intra-articular administration of corticosteroids and/or hyaluronic acid is widely used in clinical practice, although their roles are still debated [8].

Rehabilitation approaches are commonly prescribed in clinical practice and are supported by international guidelines for the management of OA, particularly exercise and assistive devices as well as physical agent modalities [9,10]. However, the effects of physical modalities on the joint environment as well as their clinical implications in the early stages of OA are poorly known. Therefore, an analysis of the literature is necessary to elucidate the mechanisms of action of this intervention in EOA, considering the huge variety of physical agents available and their applications in clinical practice. It is critical to study the effects of these interventions on not only pain management but also any changes induced to joint tissue, such as the articular cartilage and subchondral bone.

The purpose of this scoping review is to analyze the current knowledge regarding the biological effects and clinical effectiveness of physical agents in the management of patients with EOA.

## 2. Materials and Methods

This scoping review has been performed according to the PRISMA-ScR (Preferred Reporting Items for Systematic Reviews and Meta-Analyses Extension for Scoping Reviews) model [11]. The technical expert panel (TEP) consisted of eight physicians, including four physiatrists with expertise in EOA (G.L.M, D.S., G.I., A.M.), three experts in scoping review methodology (F.G., M.P., S.L.), and one orthopedic surgeon (G.T.).

The TEP investigated the biological effects and clinical effectiveness of the following instrumental physical therapy on EOA: electric stimulation therapy, pulsed electromagnetic field (PEMF), laser therapy, magnetic field therapy, extracorporeal shockwave therapy (ESWT), cryotherapy, vibration therapy, and induced hyperthermia.

### 2.1. Search Strategy

The TEP performed their research on PubMed (Public MedLine, run by the National Center of Biotechnology Information (NCBI) of the National Library of Medicine of Bethesda, Bethesda, MD, USA), with a search string combining keywords for both EOA and physical therapy modalities (see Table 1 for further details on the search strategy).

### 2.2. Study Selection

The TEP considered, for eligibility, articles published from inception to 31 December 2020, including only those in the English language (see Table 2 for further details about eligibility criteria). All data extracted from full texts and findings from included studies were qualitatively analyzed.

## 3. Results

**Figure 1:**
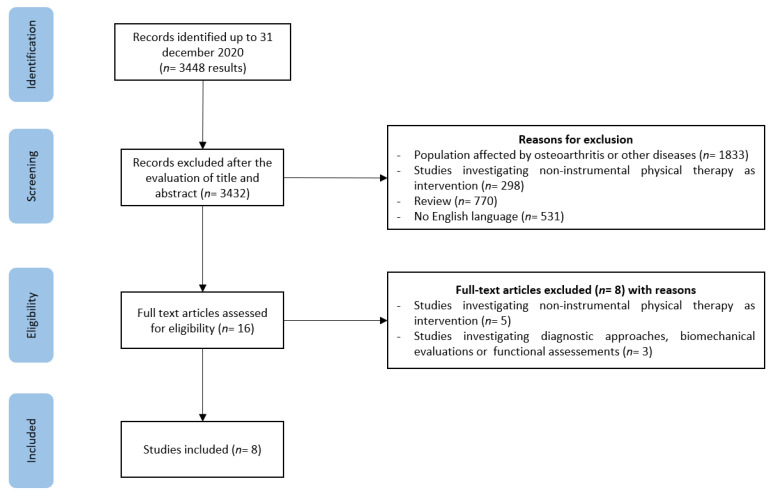
PRISMA-ScR flow diagram of the study selection process.

We initially found 3448 articles from the PubMed database. Based on the titles and abstracts and following our exclusion criteria, a total of 3432 papers were excluded. Further, eight articles were excluded after reading the full text because they did not meet our inclusion criteria. The remaining eight articles (published between April 2014 and December 2020) met the inclusion criteria (Figure 1).

All studies included in our analysis were focused on the effects of physical modalities on animal models of EKOA, except two studies investigating the efficacy and effectiveness of these interventions in patients with EKOA (Table 3). Six preclinical studies were included: five were conducted on the knees of rats and one on the knees of rabbits. One observational longitudinal study and one randomized, single-blind clinical trial were considered as clinical research. Among these studies, we found one randomized, single-blind trial concerning transcutaneous electrical nerve stimulation (TENS), three preclinical studies concerning ESWT; one preclinical study concerning low-intensity pulsed ultrasound (LIPUS); two articles concerning PEMF (one preclinical study and one prospective case series); and one preclinical study concerning whole-body vibration (WBV). No articles regarding laser therapy, cryotherapy, or other forms of induced hyperthermia were found.

### 3.1. TENS

No preclinical study investigated the effects of TENS on EOA models.

A randomized, single-blind clinical trial was conducted by Cherian et al. [12] to assess the efficacy of TENS on 23 EKOA patients (KL grades 1 and 2) in terms of pain relief, muscle strength, functional improvements, and quality of life (QoL). Patients were randomly divided into two groups: one treated with TENS for three months and another one undergoing standard conservative therapy (exercise and/or corticosteroid injections; control group).

Despite some limitations of the study, related to the small sample size and the short-term follow-up (3 months), the authors reported that TENS compared to standard therapy resulted in significant improvements in the isokinetic muscle strength of quadriceps (+5.12 ft/lb vs. −4.64 ft/lb; *p* = 0.0184), Timed-Up-and-Go test (TUGT) (−7.2 s vs. +3.9 s; *p* = 0.003), Knee Society score (KSS) (+23.2 vs. +7.2; *p* = 0.032), SF-36 score (+11.4 vs. +1.7; *p* = 0.030), and Lower Extremity Functional Scale (LEFS) score (+20.8 vs. +7.5; *p* = 0.042). Moreover, significant pain relief (baseline VAS 5 vs. 3-month VAS 2.4; *p* = 0.0027) was reported in patients receiving TENS.

### 3.2. ESWT

Three studies evaluated the effects of ESWT in animal models of EKOA. No clinical study on the role of ESWT in the treatment of EOA patients was found.

Chou et al. [13] conducted a comparative study on rats with EKOA to clarify the effects of ESWT on the articular cartilage of the medial compartment and the subchondral bone of the medial tibial plate. Fifty rats were randomly divided into five groups: Group 1 (sham group) received a simulated left knee arthrotomy without medial meniscectomy (MMx) and anterior cruciate ligament transection (ACLT); Group 2 (meniscus group) received a simulated arthrotomy of the left knee without ACLT and MMx but with ESWT applied to the medial rim of the meniscus; Group 3 (OA group) received ACLT and MMx of the left knee; Group 4 (T(M) group) received ACLT and MMx of the left knee in association with ESWT applied to the subchondral bone of the medial tibial plate; Group 5 (articular cartilage group) received ACLT and MMx of the left knee in association with ESWT applied to the articular cartilage surface of the medial tibial plate. The application of ESWT on the subchondral bone of the medial tibial plates compared to its application on articular cartilage resulted in statistically significant changes in terms of cartilage surface damage, loss of cellularity, loss of matrix staining, loss of tidemark integrity (modified Mankin score 1.60 ± 0.21 vs. 3.00 ± 0.23; *p* < 0.05), maximum extension angle of joint-surface damage (26.22 ± 4.00 vs. 57.36 ± 8.67; *p* < 0.05), bone mineral density (0.34 ± 0.03 vs. 0.29 ± 0.02; *p* < 0.05), and medial tibial plate injury (55.43 ± 8.32 vs. 87.04 ± 6.82; *p* < 0.05). The authors also analyzed the effects of ESWT on synovial tissue compared to OA and articular cartilage groups, demonstrating statistically significant differences in terms of the thickening of synovial cell lining (1.80 ± 0.20 vs. 2.60 ± 0.25 and 2.80 ± 0.20; *p* < 0.05), synovial hyperplasia (1.60 ± 0.25 vs. 2.80 ± 0.20 and 2.60 ± 0.25; *p* < 0.05), and cell infiltration (1.60 ± 0.25 vs. 2.80 ± 0.20 and 2.60 ± 0.25; *p* < 0.05), synovitis score (5.00 ± 0.40 vs. 8.20 ± 0.34 and 8.00 ± 0.40; *p* < 0.05) and IL1β expression (IL1β layer score 1.56 ± 0.24 vs. 2.22 ± 0.22 and 2.33 ± 0.20; *p* < 0.05). Moreover, the T(M) group showed significantly increased expression of chondrogenesis proteins such as TGF-β1 and DMP-1 compared to the OA and articular cartilage groups (70 ± 9.9% and 9 ± 3.9% vs. 46 ± 9.4% and 4 ± 1.9% vs. 52 ± 7.2% and 2 ± 2.5%; *p* < 0.05) as well as reduced expression of cartilage degradation enzymes such as matrix metalloproteinase 13 (MMP-13) and the A Disintegrin And Metalloproteinase with ThromboSpondin motif (ADAMTS-5) (7 ± 3.2% and 10 ± 4.1% vs. 14 ± 2.4% and 21 ± 5.2% vs. 12 ± 2.8% and 24 ± 7.9%; *p* < 0.05). Finally, authors reported that ESWT did not result in significant differences between the sham group and the meniscus group in terms of OA lesion score (0.00 ± 0.00 vs. 0.05 ± 0.05), maximum extension angle (19.12 ± 1.65 vs.19.91 ± 1.87), bone mineral density (0.35 ± 0.02 vs. 0.34 ± 0.04), and medial tibia lesion (0 vs. 0), suggesting a good safety profile of ESWT (at the dosage of 0.25 mJ/mm^2^) for treating EKOA.

Hsu et al. [14] provided the first evidence that ESWT increased the expression of several factors modulating rapid membrane signaling pathways that affect the integrity and function of chondrocytes and osteoblasts in the joint environment in EKOA. A preclinical study was conducted on 144 rats, randomly divided into 3 groups. Group 1 (normal control, NC) received neither the transection of ACLT nor ESWT; Group 2 received ACLT (OA group), whereas Group 3 underwent ACLT and received ESWT on the medial tibial plate subchondral bone (OA + ESWT). ESWT determined an overexpression of the mRNA of protein-disulfide isomerase-associated 3 (Pdia-3), a key mediator of the 1α,25-dihydroxy vitamin D 3 (1α,25(OH)2D3) non-genomic pathway, and extracellular signal-regulated protein kinase 1 (ERK1), implicated in mechanical-stimulated bone formation, compared to the OA and NC groups (*p* < 0.001). An increase in bone formation markers, such as alkaline phosphatase (ALP), osteopontin (OPG), and MMP-13, was also observed compared to OA and NC (*p* < 0.001), resulting in improved osteogenesis and an improved turnover rate of the subchondral bone in the knees affected by OA. Additionally, compared to the OA and NC groups, a decrease in cartilage matrix loss was observed in the OA + ESWT group, with higher expression of collagen II (increased by about 3 times; *p* < 0.001) and aggrecan (increased by about 20 times; *p* < 0.001). Histological analysis revealed that Group 3 had significantly lower Mankin scores compared to Group 2 at all follow-ups (2-, 4-, 8-, and 12 weeks).

The chondroprotective role of ESWT was also evaluated by Cheng et al. [15], who performed a gene processing of microRNAs (miRs) found in articular cartilage and subchondral bone following ESWT. The authors conducted a preclinical study on 30 rats undergoing ACL resection in combination with MMx (ACLT + MMx) to induce EOA-like changes in joints (OA group). Following the application of ESWT to the medial tibial plateau subchondral bone (OA + SW group), histological samples were collected and processed to identify specific miRNA activated or suppressed by the intervention and correlated with OA-related changes. ESWT stimulates or inhibits the expression of several specific miRNA for cartilage and subchondral bone, controlling genes involved in cartilage development and bone remodeling. The histological analysis showed only mild fibrillation on the surface of articular cartilage in the OA + SW group at 4 weeks. In articular cartilage, 4 miRs were found to be increased and 10 miRs were decreased in OA vs. OA + SW groups, respectively, while in the subchondral bone, 3 miRs were increased and 9 miRs were decreased in OA vs. OA + SW groups. In this group, in both articular cartilage and subchondral bone, rno-miR-181a-5p was significantly up-regulated compared to the OA group.

### 3.3. LIPUS

No clinical study about the role of LIPUS in the treatment of EOA patients was found.

Xia et al. [16] studied the chondroprotective effect of LIPUS on animal models of early and late OA. The authors conducted a preclinical study on 36 rabbits, randomly divided into 6 groups (i.e., early control, EOA, early treatment, late control, late OA, and late treatment). All groups were submitted to surgical procedures: control groups received sham operations with knee exposure, while all other groups received ACLT. Early treatment and late treatment groups were treated with LIPUS at 4 and 8 weeks after surgery, respectively. After treatment with LIPUS, the first group showed a slightly irregular cartilage surface and chondrocytes proliferation, but in the late treatment group, the cartilage damage remained almost unchanged. The Mankin score was significantly higher in the EOA group vs. the early treatment group, whereas no significant between-group difference was reported for histological changes in the late treatment vs. late OA groups. In addition, early use of LIPUS has been shown to significantly increase type II collagen expression (*p* < 0.05) and decrease MMP-13 levels (*p* < 0.05) compared to the EOA group, while no significant changes were reported in the comparison between late LIPUS vs. late OA. Authors have also investigated the effect of LIPUS on the integrin/focal adhesion kinase (FAK)/mitogen-activated protein kinase (MAPK) signaling pathway, which plays an important role in the pathogenesis of OA by targeting the ECM in articular cartilage. This study demonstrated that early application of LIPUS significantly increased the expression of integrin β1 (*p* < 0.05) and phosphorylated FAK (*p* < 0.05) and decreased the expression of ERK1/2 (*p* < 0.05) and phosphorylated MAPK 38 (*p* < 0.05) compared to the EOA group.

### 3.4. PEMF

Two papers investigated the role of PEMF in EOA (one preclinical study and one case series).

In the preclinical study of Yang et al. [17], the effects of PEMF were assessed on cartilage and subchondral bone at different stages of knee OA. Seventy-five rats were divided into five groups: the control group (sterile saline injection), a group previously treated with PEMF (in the 4 weeks preceding injections of 0.2 mg iodoacetate monosodium (MIA) to induce OA), a group early treated with PEMF (4 to 8 weeks after MIA injection), a group late treated with PEMF (from 8 weeks to 12 weeks after MIA injection), and a group with OA. Preventive treatment with PEMF was shown to preserve subchondral trabecular bone microarchitecture compared to the OA group, with significant reductions of trabecular separation (Tb.Sp) in all knee compartments (medial tibia: 111.18 ± 13.21 µm vs. 143.27 ± 13.36 µm, *p* = 0.003; lateral tibia: 120.32 ± 8.80 µm vs. 151.47 ± 4.37 µm, *p* < 0.0001; medial femur: 93.85 ± 10.46 µm vs. 119.25 ± 7.92 µm; *p* = 0.001; lateral femur: 115.03 ± 8.48 µm vs. 95.67 ± 20.84 µm, *p* = 0.002. respectively) and significantly higher bone volume fraction (BV/TV) in the medial and lateral tibia (50.11 ± 6.28% vs. 36.84 ± 3.07%, *p* = 0.001; 38.85 ± 2.89% vs. 33.71 ± 2.36%, *p* = 0.021) and in the medial femur (52.08 ± 5.34% vs. 45.34 ± 2.25%, *p* = 0.043), trabecular thickness (Tb.th) in the medial tibia and femur compartments (118.21 ± 16.98 µm vs. 87.95 ± 5.09 µm, *p* = 0.002; 104.59 ± 12.81 µm vs. 86.71 ± 7.83 µm, *p* = 0.044), and trabecular number (Tb.N) in the lateral tibia and femur compartments (4.85 ± 0.27 l/mm vs. 4.35 ± 0.12 l/mm, *p* = 0.007; 5.21 ± 0.22 l/mm vs. 4.82 ± 0.11 l/mm, *p* = 0.006). Early treatment with PEMF resulted in a significant increase of Tb.N in the medial and lateral tibia (4.18 ± 0.09 l/mm vs. 3.78 ± 0.16 l/mm, *p* < 0.0001; 4.57 ± 0.09 vs. 4.14 ± 0.17 l/mm, *p* = 0.0001) and significant reduction of Tb.Sp in the medial tibia (114.21 ± 11.42 µm vs. 135.12 ± 14.83 µm, *p* = 0.04) compared to the OA group. Tb.N of the lateral tibia was also significantly higher in the group early treated with PEMF compared to the control group (4.57 ± 0.09 l/mm vs. 4.23 ± 0.14 l/mm, *p* = 0.008). The late treatment with PEMF resulted in significant improvements of BV/TV (56.48 ± 6.03% vs. 43.54 ± 5.45%, *p* = 0.004), bone mineral density (BMD) (862.99 ± 55.37 mg/cc vs. 743.52 ± 62.77 mg/cc; *p* = 0.008), and Tb.N (4.13 ± 0.17 l/mm vs. 3.74 ± 0.24 l/mm, *p* = 0.016) and significant decreases of Tb.Sp in the medial tibia (109.46 ± 14.45 µm vs. 139.01 ± 18.80 µm, *p* = 0.019) compared to the OA group. Moreover, in the delayed PEMF group, compared to the control group, there was a significant decrease in Tb.N in the medial tibia (4.13 ± 0.17 l/mm vs. 4.55 ± 0.28 l/mm, *p* = 0.043) as well as significant increases of BMD in all knee compartments (medial tibial: 862.99 ± 55.37 mg/cc vs. 627.46 ± 44.96 mg/cc, *p* < 0.0001; lateral tibial: 762.60 ± 45.06 mg/cc vs. 583.75 ± 36.30 mg/cc, *p* = 0.001; medial femur: 657.70 mg/cc ± 61.51 vs. 845.04 ± 65.83 mg/cc, *p* = 0.007; lateral femur: 823.56 ± 41.71 mg/cc vs. 649.87 ± 40.09 mg/cc, *p* = 0.003). Furthermore, preventive and early treatment with PEMF also significantly increased the bone formation markers compared to the OA group, such as serum osteocalcin (OC) (*p* = 0.003 and *p* = 0.001, respectively), serum N-propeptide IIA of type II collagen (PIIANP) (*p* < 0.001, both), urine C-terminal telopeptide of collagen type I (CTX-I) (*p* = 0.005 and *p* = 0.004, respectively) and urine C-terminal telopeptide of collagen type II (CTX-II) (*p* < 0.001 and *p* = 0.019, respectively). Moreover, preventive and early PEMF significantly reduced cartilage degradation, whereas delayed PEMF only increased the markers of bone synthesis.

In the prospective study of Gobbi et al. [18], the effectiveness of PEMF in patients with symptomatic EKOA was investigated. Forty-eight patients were recruited, receiving PEMF for 4 h per day for a period of 45 days. Of these, only 22 patients met the study inclusion criteria (aged 30–60 years, symptomatic EKOA with KL grade 0–2) and were followed for 2 years. After the application of PEMF, improvements of pain, other symptoms (e.g., joint swelling and stiffness), participation in activities of daily living (ADL), QoL, and activity level (work and sport) were observed, and these results were maintained at 1 year, while they decreased in the 2-year follow-up. Furthermore, these results were greater in young patients (<45 years) than in those aged >45 years. At 1 year after treatment, significant improvements in KOOS Pain (52.4 ± 4.9 vs. 89.7 ± 4.4; *p* = 0.006), KOOS Symptoms (55.2 ± 5.0 vs. 87.5 ± 3.5; *p* = 0.04), KOOS ADL (53.3 ± 5.6 vs. 94.8 ± 2.9; *p* = 0.002), KOOS Sport (28.0 ± 5.9 vs. 75.4 ± 6.2; *p* = 0.001), KOOS QOL (35.6 ± 4.5 vs. 80.5 ± 4.7; *p* = 0.008), VAS (5.6 ± 0.3 vs. 1.3 ± 0.4; *p* = 0.001), and Tegner Activity scale (2.5 ± 0.5 vs. 4.5 ± 0.5; *p* = 0.002) were reported. At 2 years after treatment, the values remained higher than baseline, showing no significant improvement: KOOS Pain (52.5 ± 4.9 vs. 75.9 ± 3.6; *p* = 0.422), KOOS Symptoms (55.2 ± 5.0 vs. 72.2 ± 3.7; *p* = 0.306), KOOS ADL (53.3 ± 5.6 vs. 72.9 ± 3.9; *p* = 0.971), KOOS Sport (28.0 ± 5.9 vs. 75.4 ± 6.2; *p* = 0.503), KOOS QOL (35.6 ± 4.5 vs. 66.8 ± 6.1; *p* = 0.224), VAS (5.6 ± 0.3 vs. 2.2 ± 0.6; *p* = 0.037), and Tegner Activity scale (2.5 ± 0.5 vs. 3.8 ± 0.5). An improvement in ROM was observed both 1 year after treatment (0–131.1° ± 2.5°) and 2 years after treatment (1.2–127.2° ± 5.1°) compared to baseline (7.5–120.0° ± 4.2°). The authors also conducted a comparison of results between patients younger than 45 and those older than 45. From this comparison, statistically significant results emerged 1 year after treatment in patients younger than 45 years, particularly in terms of the Tegner Activity scale (6.1 ± 0.5 vs. 2.9 ± 0.5; *p* = 0.01)

### 3.5. WBV

No clinical study about the role of WBV in the treatment of EOA patients was found.

Wang et al. [19] investigated the effects of different frequencies (10, 20, 40, and 60 Hz) of WBV in the progression of EKOA in animal models. Forty rats were divided into five groups: a control group; a group treated with high-frequency vibrations of 60 Hz; a group treated with high-frequency vibrations of 40 Hz; a group treated with medium frequency vibrations of 20 Hz; and a 10 Hz low-frequency vibration group. In all groups treated with WBV, the treatment lasted 8 weeks. Low-frequency WBV compared to higher-frequency WBV resulted in significant reductions in the expression of IL1β (*p* < 0.001), the inducible factor by hypoxia 2-α (HIF-2 α) (*p* < 0.001), and the catabolic enzyme MMP-13 (*p* < 0.001) and increased the expression of collagen type II α 1 (COL2A1) (*p* < 0.001). Low-frequency WBV also led to a greater reduction in the degeneration of articular cartilage, assessed by the Osteoarthritis Research Society International (OARSI) grading system, compared to high-frequency WBV (*p* = 0.002 and *p* = 0.027, respectively).

## 4. Discussion

To the best of our knowledge, this is the first review that specifically addresses the effects of physical agent modalities in EOA. According to PRISMA-ScR [11], our paper aims to synthesize evidence and identify gaps on a specific topic (how physical agents might work in EOA) “from a body of knowledge that is heterogeneous in methods or discipline”, considering that EOA is still a debated topic. Indeed, our review also suggests that the studies included did not use any of the diagnostic criteria proposed for EOA so far [4,5,6].

This is a complex disease to deal with, considering that consensus about its definition, diagnosis, and treatment is still debated. Its management is even more intricate due to the high variability of clinical and instrumental findings. Therapeutic approaches recommended for managing OA [9,10], such as non-surgical interventions, including physical therapies, are usually also first-line interventions for EOA [20]. In clinical practice, physical agent modalities are widely used alone or in combination with other conservative treatments in all phases of OA [21]. However, their use is supported by a few studies with methodological limitations, particularly in dosage information, and their effects are usually based on short-term pain relief only. Moreover, for some physical agent modalities (i.e., TENS), recent international guidelines have recommended against their use in patients with knee or hip OA. Despite poor evidence available, physical therapies are widely used as adjuvant interventions for OA, also considering their optimal safety profile [21,22]. Another key issue concerns the timing of the intervention of physical agents in OA, as suggested by some experimental data. For example, LIPUS seems more effective in the early phases than in the late phases of joint degeneration.

As demonstrated by our review, several physical modalities have been studied for the treatment of EOA, such as TENS, ESWT, LIPUS, PEMF, and vibration therapy, although these interventions have been predominantly investigated in preclinical studies, all aimed at the treatment of EKOA. It should be emphasized that the clinical implications of the findings derived by the preclinical studies should be considered with caution, considering that in animal models, the EOA is mainly due to trauma (ACL injuries and/or meniscus injury). Moreover, the clinical studies included in our review used different diagnostic criteria for EOA (Kellgren–Lawrence grade 1 or 0–2) [12,18].

According to our scoping review, TENS showed pain-relieving action along with beneficial effects on joint function, quadriceps strength, physical performance, and QoL in patients with EKOA [12]. The functional benefits of TENS has opened interesting scenarios, supporting its use in combination with therapeutic exercise. Indeed, exercise is a core strategy in all stages of OA [10] and has been demonstrated to be also effective in EKOA in middle-aged adults, including high-risk populations (i.e., athletes) [23,24].

Emerging evidence supports ESWT as non-invasive therapy for OA [25], whereas data about the effectiveness of this physical modality for patients with EOA are lacking. Chou et al. [13] have shown on animal models that ESWT has protective effects for joint tissues (i.e., articular cartilage, synovium, subchondral bone), delaying the progression of OA. These effects were superior if ESWT was applied directly to the subchondral bone rather than to articular cartilage.

Cheng et al. [15] performed a gene analysis of miRNAs expressed in joint tissues after an application of ESWT, revealing rather conflicting data that do not allow us to reach clear and definitive conclusions regarding the role of ESWT in EKOA.

Significant up-regulation of miR-181a-5p was observed both in articular cartilage and subchondral bone in rats with EKOA receiving ESWT compared to the EKOA group [15]. It has been reported that the up-regulation of miR-181a-5p increases oxidation of the ECM by reducing the expression of selenoprotein glutathione peroxidase 1 (GPX1) and 4 (GPX4) through the inhibition of their target, selenocysteine insertion sequence binding protein 2 (SBP2), finally resulting in cartilage damage [26]. However, Cheng et al. [15] reported only negligible damage of the articular surface, with slight fibrillation in rat knees treated with ESWT.

The protective action of ESWT on the joint environment in EKOA could be attributable to the increased expression of mediators involved in the membrane signaling pathway, such as Pdia-3, ERK1, OPG, ALP and MMP-13, after only 2 weeks, resulting in favorable histological findings [14]. Pdia-3 is a protein that mediates the membrane response to 1α, 25 (OH) 2D3, and it is involved in the regulation of protein kinase C (PKC) activation and the release of prostaglandin E2 (PGE2), following stimulation by phospholipase A2 (PLA2). In this way, Pdia-3 regulates the transcription of genes related to bone mineralization through the phosphorylation of transcription factors such as ERK 1/2 in cells with mitogenic functions, similar to osteoblasts. ERK-1 is a kinase that acts as a mediator for the differentiation and proliferation of osteogenic cells. Once activated by Pdia-3, following the application of ESWT, ERK-1 migrates to specific nuclear targets, causing an increase in bone formation in the areas of damage. Therefore, by modulating the expression of Pdia-3 in the subchondral bone, ESWT improves subchondral bone remodeling.

LIPUS stimulates the proliferation of chondrocytes and prevents cartilage damage in EOA through the integrin/FAK/MAPK pathway with an increase in type II collagen and a reduction in the expression of MMP-13 [16]. This occurs because the application of LIPUS in an early stage of OA determines a greater expression of β1 integrin and phosphorylated FAK and down-regulation of the expression of ERK1/2 and phosphorylated p38. On the contrary, in advanced OA, this intervention resulted in the down-regulation of β1 integrin and phosphorylated FAK and the up-regulation of ERK1/2 and p38, which might negatively affect the disease course. These findings indicate that the time schedule of physical modalities might play the main role in the management of OA.

PEMF seems a promising therapeutic option for EKOA, as it preserves subchondral trabecular bone microarchitecture, prevents subchondral bone loss, and increases bone and cartilage synthesis. These protective effects on the subchondral bone were more pronounced in animal models previously treated with PEMF compared to early or delayed interventions after the induction of OA [17]. From a clinical perspective, the effectiveness of PEMF has been investigated in a prospective study, including patients with EKOA. This intervention was demonstrated to be effective in terms of pain relief and improvement of knee function, physical performance, and QoL at both 1- and 2-year follow-ups, particularly in patients younger than 45 years, although a significant worsening of outcome measures between the first and the second year was observed [18]. These findings suggest the need to repeat PEMF after a certain period. Moreover, the age of the patient with EKOA can play a critical role in the effectiveness of PEMF, with better results generally obtained in patients at a younger age.

Experimental data suggest that exposure to low-frequency WBV has protective effects on the knee joint as it counteracts the degeneration of articular cartilage in rats [19]. Its main observed effects were the reduction of the expression of cartilage catabolic factors (HIF-2α, MMP-13) and inflammatory mediators (i.e., IL1β), along with the increased expression of COL2A1, resulting in favorable histological findings.

Starting from preclinical studies conducted on animals, further implementation through research on clinical studies conducted on humans is essential to be able to identify a standard method based on exact dosages and time parameters.

The main limitation of our review is the search strategy, including only one (although the most used) database (i.e., PubMed), which might be a selection bias. Another limitation is due to the prevalence of preclinical scientific studies conducted on small animals (rats and rabbits), whose anatomy and physiology of the knee differ from that of humans. This could decisively influence the reproducibility of the results obtained in future studies in humans, where, in addition to the different dimensions, there are different anatomical and physiological characteristics. Further limitations are the lack of information on the safety of some physical therapies (e.g., WBV), the appropriate dosage recommended for humans, and the lack of data on pain and disease progression in most animal studies.

## 5. Conclusions

Identification of EOA is challenging because different diagnostic criteria have been proposed in the last decade. This issue must be solved to better define the role of the available therapeutic options for OA. Our review contributes to increasing knowledge about the mechanisms of action of several physical therapies in EKOA, suggesting their role in modifying disease progression, mainly thanks to its action on the subchondral bone and through gene modulation. In this context, experimental data suggest that the effects of physical modalities are affected by the timing of the intervention with physical modalities. In clinical practice, these interventions are widely used, with weak scientific support, particularly concerning the limited data available from clinical studies.

## Figures and Tables

**Table 1:** Search Strategy.

(“Electric Stimulation Therapy” [Mesh] OR “Pulsed Electromagnetic field” OR “Low-Level Light Therapy” [Mesh] OR “Laser Therapy” [Mesh] OR “Magnetic Field Therapy” [Mesh] OR “Extracorporeal Shockwave Therapy” [Mesh] OR “Hyperthermia, Induced” [Mesh] OR “Cryotherapy” [Mesh] OR “Vibration therapy” OR “Whole Body Vibration” OR “Physical Therapy Modalities” [Mesh]) AND (“Osteoarthritis” [Mesh] OR “Osteoarthritis, Spine” [Mesh] OR “Osteoarthritis, Knee” [Mesh] OR “Osteoarthritis, Hip” [Mesh] OR “Early Osteoarthritis”)

**Table 2:** Eligibility criteria.

Inclusion Criteria
- English language - Reference period: from inception to 31 December 2020 - Study design: preclinical and clinical studies, including case reports, clinical trials, and observational studies. - Studies including instrumental physical therapies for patients with EOA at any joint as intervention
**Exclusion Criteria**
- Books and documents, meta-analyses, reviews, systematic reviews, letters to the editor - Population affected by osteoarthritis. - Articles written in other languages. - Studies investigating non-instrumental physical therapies as intervention

**Table 3:** Characteristics and findings of the included studies evaluating the effects, efficacy, and effectiveness of physical agent modalities in early osteoarthritis.

Author, Year	Physical Therapy Modality	Study Design	Sample Size: Total (Group)	Administration	Main Findings
Cherian et al., 2015 [12]	TENS	Prospective, randomized, single-blinded trial, including EKOA patients (Kellgren–Lawrence grade 1)	*n* = 23 TENS group = 13 Control group = 10	TENS: device included in a brace to wear for the entire day Pulse waveform: asymmetric, biphasic, and simple modulated Pulse rate: 12 s intervals of grouped pulses Voltage current: 48–400 μs at 50% peak amplitude Duration of the intervention: 3 months Control: self-directed exercise therapy and/or corticosteroid injections	TENS significantly improved quadriceps strength, TUGT, objective KSS score, LEFS, and physical component of SF-36 compared to controls.
Chou et al., 2019 [13]	ESWT	Preclinical study on EKOA rat model (ACLT + MM)	*n* = 50 5 groups (10 rats in each group)	Group I Sham: arthrotomy of left knee Group II Meniscus: arthrotomy and ESWT applied to the medial edge of the meniscus Group III OA: anterior cruciate ligament transacted (ACLT) and medial meniscectomy (MMx) Group IV T(M): ACLT and MMx of left knee and ESWT applied to the proximal medial tibia plateaus Group V Articular Cartilage: The animals received ACLT and MMx of left knee and ESWT applied to the articular cartilage surface of the proximal medial tibia plateaus One ESWT at 1-week post-surgery with ultrasound guidance ESWT: 800 impulses at 0.25 mJ/mm	ESWT applied to the subchondral bone has protective effects for articular cartilage, synovium, and subchondral bone.
Hsu et al., 2017 [14]	ESWT	Preclinical study on EKOA rat model (ACLT)	*n* = 144	Group I: normal control (NC) Group II: EKOA induced by ACLT Group III: EKOA induced by ACLT receiving ESWT (800 impulses at 0.18 mJ/mm^2^, 4 Hz frequency) to the subchondral bone of the medial tibia plate. 12 rats in each group were sacrificed at 2, 4, 8, and 12 weeks post-surgery. Of the 12 rats at 2 weeks post-surgery, the articular cartilage and subchondral bone of tibia of 6 rats were used for proteome analysis and the joints of another 6 rats for immunohistochemistry analysis.	ESWT might affect chondrocytes’ and osteoblasts’ functions in the joint environment by modulating several factors of the rapid membrane signaling pathway, including Pdia-3, ERK1, OPG, ALP, and MMP13. These factors significantly increased at 2 weeks post-treatment, resulting in favorable histological changes.
Cheng et al., 2016 [15]	ESWT	Preclinical study on EKOA rat model (ACLT + MM)	*n* = 30	Group I: sham Group II: OA Group III: OA + ESWT applied on the subchondral bone of the medial tibia plateau One ESWT at 1-week post-surgery. ESWT: 800 impulses at 0.22 mJ/mm and 4 Hz frequency Histological and miRNA analyses were performed after 4 weeks. A set of 729 miRNAs expressed in cartilage and subchondral bone was obtained.	ESWT induced expression of miRNA to control genes correlated with cartilage development and bone remodeling. In the ESWT group, the articular surface damage was not obvious and only mild fibrillation was observed.
Xia et al., 2015 [16]	LIPUS	Preclinical study on rabbit EKOA model (ACLT)	*n* = 36	Group I: early control (6) Group II: early osteoarthritis (6) Group III: early treatment (6) Group IV: late control (6) Group V: late osteoarthritis (6) Group VI: late treatment (6) The early and late treatment groups were exposed to low-intensity pulsed US 4 and 8 weeks after surgery	LIPUS protects cartilage from damage in early-stage osteoarthritis via the integrin/FAK/MAPK pathway
Yang et al., 2017 [17]	PEMF	Preclinical study on EKOA rat model (induced by low-dose of MIA)	*n* = 75	Group I: OA (30) Group II: pre-emptive PEMF (10) from day 0 to end of week 4 Group III: early PEMF (10) from week 4 to end of week 8 Group IV: delayed PEMF (10) end of week 8 to end of week 12 Group V: control (15) After 1 week, rats in OA and PEMF groups were injected with 0.2 mg MIA through the infrapatellar ligament of the right knee only once. MIA was dissolved in sterile physiologic saline and administered in a 50 mL microsyringe. Control rats received a 50 mL sterile physiologic saline injection. PEMF: Frequency of 75 Hz Intensity of 1.6 mT Duration: 2 h/day for 1 months during the activities of daily life	Pre-emptive and early PEMF treatment significantly increased bone and cartilage synthesis and decreased bone and cartilage degradation Pre-emptive PEMF treatment has a more beneficial effect on subchondral trabecular bone microarchitecture. Delayed PEMF treatment only increased bone synthesis The time point of treatment initiation is crucial for treating OA
Gobbi et al., 2014 [18]	PEMF	Prospective study (EKOA patients Kellgren–Lawrence grade 0–2)	*n* = 22	PEMF (4 h per day) for 45 days The maximum intensity of the magnetic field was 1.5 mT and the frequency was 75 Hz. 1- and 2-year follow-up.	PEMF reduced symptoms (pain and joint swelling) and improved knee function and activity level in EKOA patients at the 1-year follow-up, especially in young patients. These effects decreased at 2-year follow-up
Wang et al., 2020 [19]	WBV	Preclinical study on EKOA rat model (induced by 0.15 mL mixture of 4% papain and 0.03 mmol/L l-cysteine into knee joint cavity)	*n* = 40	Group I: sham control (SC) Group II: high frequency 60 Hz (HV1) Group III: high frequency 40 Hz (HV2) Group IV: middle frequency 20 Hz (MV) Group V: low frequency 10 Hz (LV) WBV (peak acceleration 0.3 g): 40 min/day and 5 days/week	WBV could alleviate the degeneration of articular cartilage. WBV regulates related gene expression at both mRNA and protein levels. HIF-2α could be a therapeutic target. The effect of WBV seems frequency-dependent: lower frequency shows better effects

**Abbreviations.** TENS: transcutaneous electrical nerve stimulation; ESWT: extracorporeal shockwave therapy; EKOA: early knee osteoarthritis; KSS: Knee Society score; TUGT: Timed-Up-and-Go test; LEFS: Lower Extremity Functional Scale; SF-36: Short Form Health Survey-36 score; VAS: visual analog scale; PEMF: pulsed electromagnetic field therapy; ROM: range of motion; ACLT: anterior cruciate ligament transacted; MMx: medial meniscectomy; MIA: monosodium iodoacetate; Pdia-3: protein-disulfide isomerase-associated 3; OA: osteoarthritis; WBW: whole-body vibration; SC: sham control; HV1: high frequency 60 Hz; HV2: high frequency 40 Hz; MV: middle frequency 20 Hz; LV: low frequency 10 Hz; HIF-2α: hypoxia-inducible factor-2α; US: ultrasound; OPG: osteopontin; ALP: alkaline phosphatase; MMP-13: matrix metalloproteinase 13; qPCR: quantitative polymerase chain reaction.
